# PMMoTo: A Porous Media Morphology and Topology Toolkit

**DOI:** 10.21105/joss.09711

**Published:** 2026-02-20

**Authors:** Timothy M. Weigand

**Affiliations:** 1 Department of Environmental Sciences and Engineering, University of North Carolina at Chapel Hill, North Carolina, United States of America

## Abstract

The Porous Media Morphology and Topology Toolkit (PMMoTo) is a software tool designed to help researchers analyze and understand porous structures and how their features influence behavior at larger scales. A porous medium is any solid material that contains pores, for example, soil, membranes, skin, and many other natural and engineered materials. In fact, porosity is often a function of spatial scale; all materials have some degree of porosity, you just need to zoom in. The morphology (i.e. the shape and structure) and topology (i.e., the connectivity and spatial relations) of a porous structure govern larger-scale behavior. By characterizing these structures, PMMoTo enables researchers to better understand emergent behaviors and develop more predictive models of porous systems.

## Statement of Need

While several excellent packages, such as PoreSpy (Gostick et al., 2019), support the analysis of porous structures, PMMoTo is distinct in two key regards: (1) PMMoTo focuses on computationally generated, often time-varying, porous structures rather than experimental or image-based data; and (2) PMMoTo is designed to handle large and/or periodic domains. Using computationally generated porous structures avoids the limits of experimental imaging resolution but introduces the need for grid resolution studies and numerical assessment of resolution effects. Highly resolved systems are often required for accurate analysis, making efficient computational methods essential. To ensure scalability and efficiency, performance-critical components of PMMoTo are implemented in C++ and Cython. For many research applications, especially those involving upscaling, large and periodic domains are required to obtain statistically representative estimates of larger-scale properties. To handle these requirements, PMMoTo is optimized for distributed-memory systems using the Message Passing Interface (MPI). Additionally, PMMoTo supports the specification of boundary conditions, including periodic boundaries, to match requirements for simulations of porous structures.

PMMoTo includes parallel implementations of common morphological operations, with communication managed to ensure correctness for all allowable boundary types. Parallelism is accomplished either by modifying and extending the underlying algorithms, such as with the Euclidean distance transform (Silversmith & Hilei, 2024) and morphological operators like dilation and erosion, or by applying exact corrections, as in the case of the connected component analysis (Silversmith, 2021). Additional tools include parallelized implementations of Minkowski functionals (Boelens & Tchelepi, 2021) and other widely used analysis methods, such as averages, histograms, and quantities relevant for upscaling approaches. PMMoTo also includes morphological approaches for evaluating multiphase equilibrium states in porous structures (Hilpert & Miller, 2001; Schulz et al., 2007).

## Research Application

**Figure 1:**
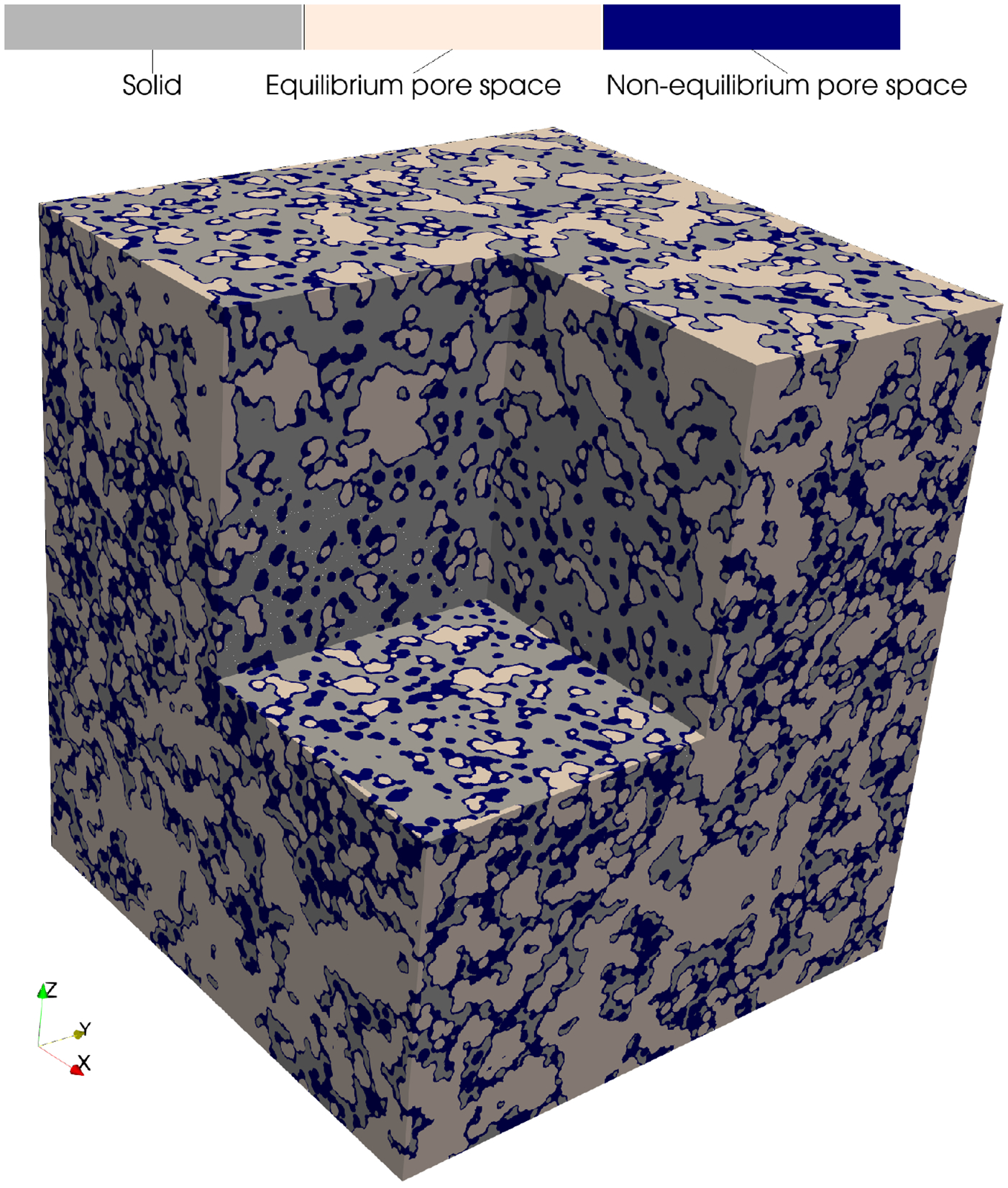
Comparison of porous structures under different assumptions about atom size for simulated polyamide membranes. PMMoTo provides tools to generate and analyze such porous structures. (Weigand et al., 2025).

Recently, PMMoTo was used to generate and analyze highly resolved probabilistic porous structures derived from molecular dynamics (MD) simulations (Vickers et al., 2022; Weigand et al., 2025) using a voxel resolution of 0.05 Å to produce a domain with 49.5 billion voxels (Figure 1). These simulations were conducted to investigate water transport through reverse osmosis polyamide membranes and assess assumptions regarding the nature of the polyamide structure. With support from observed simulation data, ephemeral water-accessible pathways (i.e. transiently connected pathways that span form the inlet to the outlet of the membrane) were identified. While it is well understood that polyamide membranes are water permeable, PMMoTo enables a novel computational approach that links atomistic simulations to larger-scale observations through morphological and topological analysis of the porous structure. This approach aims to improve our understanding of transport across spatial scales and to support the design of improved membranes.
